# Is Deep Brain Stimulation Associated With Detrimental Effects on Cognitive Functions in Patients of Parkinson's Disease? A Systematic Review

**DOI:** 10.7759/cureus.9688

**Published:** 2020-08-12

**Authors:** Ankush Maheshwary, Divya Mohite, Janet A Omole, Karandeep S Bhatti, Safeera Khan

**Affiliations:** 1 Neurology, California Institute of Behavioral Neurosciences and Psychology, Fairfield, USA; 2 Medicine, Mrs. Khushbir Kalra's Memorial Hospital, Amritsar, IND; 3 Internal Medicine, California Institute of Behavioral Neurosciences and Psychology, Fairfield, USA

**Keywords:** deep brain stimulation, subthalamic nucleus, cognition, levodopa, verbal fluency, hand tremors, movement disorder, huntington disease, attention, parkinson's disease

## Abstract

Deep brain stimulation (DBS) is a rapidly evolving procedure with its application in multiple fields of neurology, but it is most prominent in Parkinson's disease (PD). Through electrode implantation in different areas of the brain, it brings a favorable change to the motor symptoms to the magnitude that none of the medications have been able to, but the effect on cognition of the patients is still unknown. We did a comprehensive search through PubMed and Cochrane databases and conducted a systematic review by following the PRISMA guidelines. Inclusion criteria were studies conducted only in PD patients, after the year 2008. The studies published in languages other than English were excluded. Thirteen studies, including randomized and non-randomized controlled trials, observational studies, and meta-analysis, were analyzed in detail. The results showed a declining trend in verbal fluency and attention domains of cognition, while other functions remained unchanged. The decline was significant but not enough to impact the quality index in patients. DBS is associated with worse performance in verbal fluency and attention, and there is a further need for studies focusing on these domains with long-term follow-up. The overall cognitive profile was not affected significantly.

## Introduction and background

Parkinson's disease (PD), first described by James Parkinson (1755-1824) as “paralysis agitans” in his “Assay on the Shaking palsy,” is at present the second most crucial neurodegenerative disease in the elderly population [1], second only to Alzheimer's disease. The average prevalence of PD rises steadily with age, from 41 per 100,000 in the fifth to 1903 per 100,000 in the ninth decade [2]. Pathologically it is classified as synucleinopathy, associated with the loss of dopaminergic neurons primarily in the substantia nigra, but other brainstem neurons are also involved [3]. Although PD was earlier classified as a pure motor disorder with a tetrad of rigidity, bradykinesia, tremors, and postural instability, it is now considered a complex disease, including psychological, sensory, autonomic, cutaneous abnormalities [4]. There is a myriad of the neurobehavioral defects in advanced PD like depression, dementia, bradyphrenia, apathy, fearfulness, anxiety, emotional lability, social withdrawal, visual-spatial impairment, sleep disturbance, and psychosis [4]. 

Dopaminergic medications have been the mainstay of symptomatic therapy for motor symptoms of PD [5], with levodopa being the gold standard for the past 50 years [6]. It is often used in combination with dopaminergic agonist (DA) and monoamine oxidase-B inhibitors. However, the long-term use of levodopa has been associated with fluctuations in motor response with a severe impact on the patient's quality of life (QOL) [7]. Its unique pharmacodynamic and pharmacokinetic properties like discontinuous drug delivery, short half-life, poor bioavailability, and narrow therapeutic window are all crucial for such fluctuations [8]. 

Motor fluctuations are uncommon with DA monotherapy, and the occurrence seems to be delayed when levodopa is added later to agonist monotherapy. However, no studies have assessed whether initiation of levodopa with dopamine therapy gives long-term benefits concerning the development of dyskinesias [9]. Also, the use of DAs is associated with significant side effects like hallucinations, somnolence, leg edema [10], and impulse control disorders like gambling, compulsive sexual behavior, compulsive buying, or binge eating [9].

Deep brain stimulation (DBS), in its most recent iteration, has a short history of a couple of decades, even though intermittent, chronic stimulation via external electrodes has been used for various movement disorders including PD from the 1960s [11]. Tröster and colleagues in France studied the thalamic stimulation of PD tremors in the 1980s and published their outcomes of a DBS trial in the 1990s [11,12]. Modern DBS devices consist of an implantable pulse generator (akin to a pacemaker), usually implanted below the clavicle connects via an extension that runs beneath the skin of the neck to the head. The device is linked to the leads which have multiple electrodes (contacts) and are implanted in the target structure, that can be the ventral-intermediated nucleus of the thalamus (Vim) (though this procedure is rarely done anymore in PD because it alleviates only tremors), the internal segment of globus pallidus (GPi) and the subthalamic nucleus (STN) [2,10].

Universally accepted for its potentially curative effects on the motor symptoms, it has been previously tried on patients only when the medical therapy failed or to resolve the symptoms related to medical treatment. Recent randomized controlled trials (RCT) have proven that DBS, even at earlier stages of PD, is superior to medical therapy [13]. It has an overall positive impact on the QOL and decreases motor complications; however, the cognitive effects on appropriately selected PD patients are still unclear, with many studies producing contrasting results [14]. 

Its effects on the neural circuitry could partially explain the influence of DBS, mainly the cortico-striato-thalamocortical loops, but nothing has yet been proven [12]. We now compile data on the cognitive maladjustments of DBS in PD patients after reviewing different studies. We look at various aspects of cognitive changes due to constant subthalamic stimulation only and not the after-effects of stereotactic neurosurgery or electrode placement. Hence, we can justify if DBS is associated with detrimental effects on cognition in PD.

## Review

Methods

We have conducted a systematic review according to PRISMA [15] guidelines. We did a comprehensive search in PubMed and the Cochrane Central database. We used Keywords like “deep brain stimulation,” “Parkinson's disease,” “cognitive effects,” and Boolean terms “AND” and “OR” were also used. The search rendered 522 results in PubMed and 98 results in the Cochrane library database after we applied appropriate filters. The studies were then selected based upon the following recommendations.

Studies accepted for inclusion were: (a) those having patients diagnosed with idiopathic Parkinson's disease as the study population; (b) have been published between 2008 and 2019; (c) published in indexed and peer-reviewed journals; (d) published in the English language only; (e) evaluated any one of the cognitive features, including executive functions, attention, and memory.

**Figure 1:**
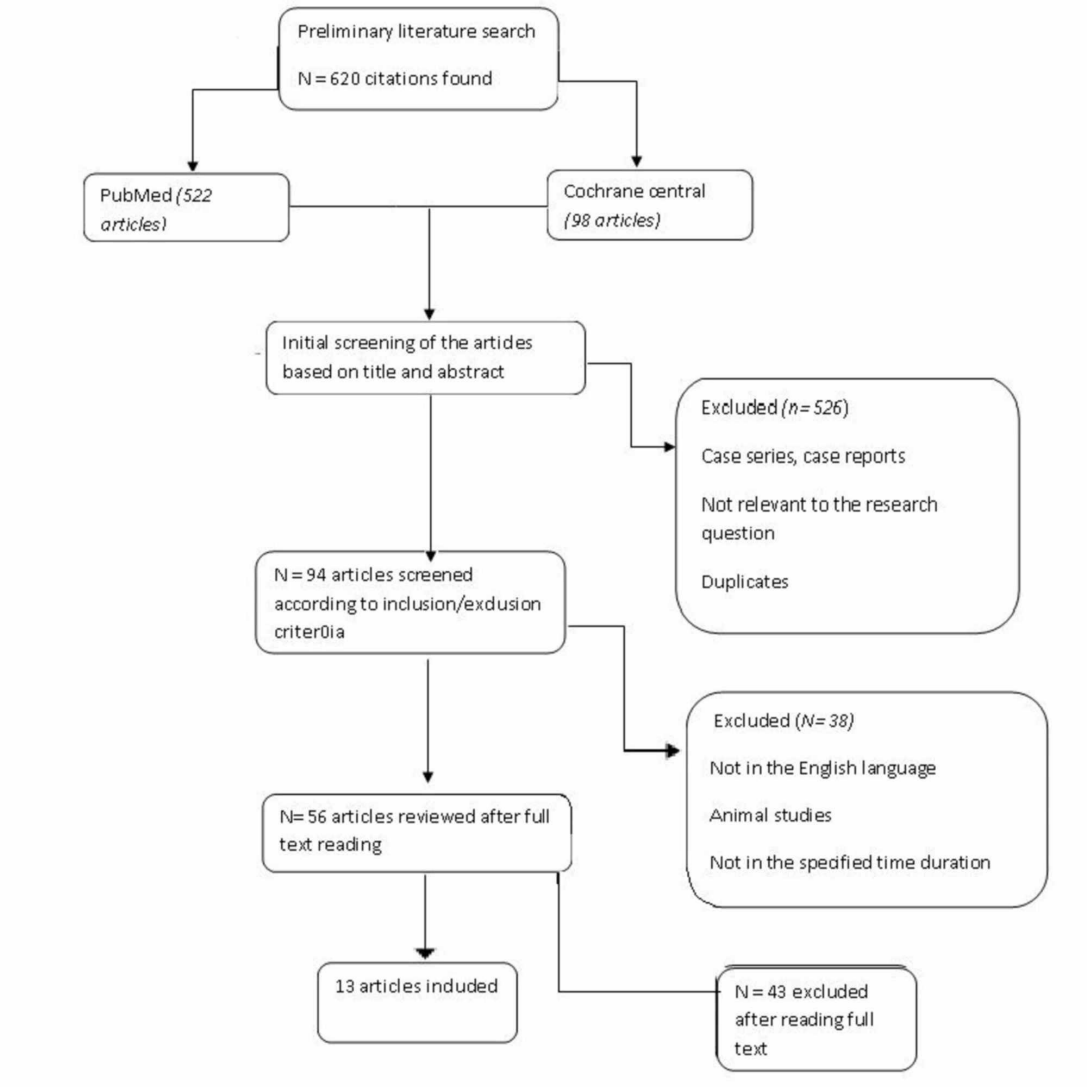
Flow chart depicting the selection of studies by appropriate use of PRISMA guidelines for systematic review

Exclusion criteria include: (a) studies published in regional languages other than English; (b) that included a comparison between STN and GPi stimulation; (c) materials from grey literature. We did a quality check for each of the selected studies and finally narrowed down the result to 13 studies (Figure 1).

Results

Among the 50 articles selected from the initial screen, we chose 13, out of which five were RCTs, six were non-randomized or observational studies, and two were systematic reviews. Both the meta-analysis studies included studies conducted before 2008. We deliberately selected studies that assess different patient characteristics as a predictor of cognitive outcomes. One of the studies specifically included early-stage PD patients as candidates for DBS [16]. Another study used a reliable change index methodology and included patients with mild cognitive impairment (MCI) as well as healthy controls for comparison. Almost all the studies had motor functioning as their primary outcome. They reported a drastic improvement in motor symptoms and fluctuations, amounting to a better overall QOL, but the results regarding cognitive issues were varied. The underlying table gives a better description of all the studies selected for review (Table 1).

**Table 1:** Demographic and clinical aspects of patients in the selected studies PD: Parkinson's disease, DBS: deep brain stimulation, STN: subthalamic nucleus, RCT: randomized controlled trial, NRCT: non-randomized controlled trial, QOL: quality of life, BMT: best medical treatment.

Study	Sample Size	Study Design	Target (STN/GPi)	Age (DBS Group/Control)	Duration of Illness (DBS Group/Control)	Follow- up	Conclusion
Troster et al. [17]	136	RCT	B/L STN	60.6 (8.5)/59.5 (8.2)	12.1 (4.9)/11.7 (4.1)	12 months	Good cognitive safety profile except for verbal fluency and attention.
Schuepbach et al. [13]	251	RCT	B/L STN	52.9±6.6/52.2±6.1	7.3±3.1/7.7±2.7	24 months	Neurostimulation was superior to medical therapy in the early motor complications in PD.
Weaver et al. 2009 [18]	255	RCT	STN/GPi	62.4 (8.8)/62.3 (9.0)	10.8 (5.4)/12.6 (5.6)	Six months	DBS was superior to BMT in improving the QOL but was associated with more adverse events.
Witt et al. [19]	123	RCT	B/L STN	60.2 (7.9)/59.4 (7.5)	13.8 (6.3)/14.0(6.1)	six months	The decline in verbal fluency and attention with no change in global cognition and mood.
Tramontana et al. [16]	30	RCT	B/L STN DBS	60±6.8/60±7.0	2.2±1.4/2.1±1.1	24 months	DBS in the early stages of PD has a better cognitive profile than in advanced PD.
Foki et al. [20]	43	Observational	STN	62.9±6.5/60.0±10.2	_	12 months	10% showed a decline in only the verbal fluency domain of cognition.
Zangaglia et al. [21]	65	NRCT	STN	58.84±7.7/62.52±6.82	11.84±5.07/9.97 ±4.86	36 months	STN has a safe cognitive profile in all domains but worsening fluency and transient executive dysfunction.
Saez-Zea et al. [22]	21	NRCT	B/L STN	54(14)/62(10)	12(2)/5(6)	6 months	The good cognitive outcome with insignificant changes can be attributed to medication reduction.
Castelli et al. [23]	58	NRCT	B/L STN	60.6 (6.7)/60.2 (6.6)	15.3(5.1)/15.6(5.2)	12 months	Phenomic verbal fluency declined after one-year post-DBS, and 15% of patients showed a significant cognitive decline after one year.
Kishore et al. [24]	45	Observational		55.4±10.9	11.1±5.5	5 years	An insignificant impact of DBS on patients with advanced PD in terms of cognition and overall QOL.
Fasano et al. [25]	16	Observational	B/L STN	56.9 ±7.2	13.7±4.8	8 years	DBS was a safe procedure in terms of cognition and behavior on long-term follow-up.
Martinez-Martinez et al. [26]	12 studies (430)	Meta-analysis	_	_	_	_	After meta-analysis, significant changes only in the verbal fluency were found.
Xie et al. [27]	Five studies (192/122)	Meta-analysis	_	_	_	_	The decline in global cognition with subtle changes in memory, fluency, and executive functions.

Discussions

It is a well-known fact that DBS in patients with PD brings about a significant change in the motor outcomes; predictable motor benefits on post-implantation follow-up were noted by all the studies included in the review. There was a drastic reduction in motor fluctuations experienced in “off medication” and “on medication” state in almost all the patients. Medication dosage declined as well, and patients could perform daily activities and executive motor functions with greater ease. In PD, the progression of MCI to overt dementia is already high, with the prevalence of dementia being 20-70% [28,29]. The interaction between MCI and DBS is less understood, and the results of cognitive changes are often variable as mixed results are obtained in various studies. 

Changes in Verbal Fluency

Troster et al., in their controlled trial, compared the pre-DBS and post-DBS cognition in PD patients after dividing the DBS group into two subgroups. These were the active stimulation (stimulation initiated immediately after the electrode implantation) and delayed stimulation (electrode stimulation commenced after three months). They noted a decline in letter verbal fluency in both subgroups, but it was much more significant in the active stimulation group one year after surgery [17]. A similar hypothesis was drawn by Kishore et al., who followed up the patients after the surgery for an extended period of five years and gathered that out of the nine patients who showed a new-onset decline in verbal fluency tasks, six patients were identified at one-year follow-up. The decrease was mild and insignificant and worse at a one-year follow-up and persisted at the same level throughout the duration [24]. Both the studies hypothesized that some “microlesion effect” in the area surrounding the implanted electrodes could explain the functional decline [17,24]. 

Witt et al. compared patients in the DBS group to the patients receiving the best medical treatment (BMT) and found out that the DBS group performed worse in the Mattis dementia rating scale. They proved that this downfall was due to the effects of semantic and phenomic verbal fluency subscale scores and repeated the test after excluding the verbal fluency component from the scale. This led to the diminution of the decline from 12% of patients in the DBS before, to 5% after exclusion. The percentage of patients with Mattis dementia rating scale decline in the BMT group remained the same at 6% [19]. They contributed to the fact that executive dysfunctions were related to intervention and not the progression of the disease. This moderate decline in verbal fluency did not affect the QOL of PD patients after surgery, even in the communication and cognition subscale of PD questionnaire- 39 (PDQ- 39) scores [19]. Foki et al. discovered the deterioration in verbal fluency at least in some patients after 12 months of DBS using a different approach of evaluation, by using a neuropsychological test battery Vienna-short version for testing cognition and analyzing the result using reliable change index (RCI) methodology. A unique feature of the study was comparing PD-DBS patients and patients with MCI and healthy controls simultaneously, which gave a better insight into the fact that these changes in cognitive domains are not related to changes due to healthy aging [20]. 

Castelli et al. employed the single case analysis method to show that there was significant cognitive deterioration in only four patients (15%) out of the 27 who received DBS. After running the independent samples nonparametric test, they correlated this cognitive decline to having worse motor symptoms at the baselines, i.e., before the surgery. The decline was seen in phenomic verbal fluency only and was not associated with the disease's progression. Other cognitive domains, like semantic verbal fluency and memory functions, were preserved [23]. Fasano et al. presented a worse verbal fluency outcome at the end of eight years. The patients in their controlled trial who performed worse in fluency tasks also had worsening postural instability that suggested some interplay between axial motor deterioration and cognition [25].

On the contrary, Saez-Zea et al. noted only a trend towards more deterioration of verbal fluency after DBS. Still, it did not differ significantly from the control group. They detected a direct correlation between the reduction in levodopa equivalent daily dosage and deterioration in phenomic verbal fluency, as they did not change the medication in the control group, and no such decline was detected in control, so they suggested that changes in the medication regime might predict the underlying declines [22]. Their study showed that stimulation of the STN and its surrounding structures might have induced functional changes in the neural network controlling cognition. However, interindividual variability was high, and the change was dependent upon individual factors such as PD phenotype, location of electrode placement, and medication change [22]. One of the most prominent clinical trials included in this review, Schuepbach et al. randomized 251 patients suffering from PD into DBS and BMT group and discovered no significant decline after 24 months but an increment in the overall cognition after DBS. However, this study did not look at the individual aspects of cognition [13]. The DBS group had worse performance in the PDQ-39 communication subscale. Still, overall improvement was evident in QOL by 26% at the end of 24 months after stimulation compared to a 1% decline in the BMT group [18]. Similar results were noted by other studies like Tramontana et al. [16], as they noted that the verbal fluency decline was only transient, and functionality returned to the baseline after some time. The stimulation-related disruption of the frontal-temporal loop, which has been attributed to be involved in verbal fluency, was ruled out as they tested the patients in the DBS group in “off stimulation” and “on medication” which gave the same results in cognitive functions [16]. We included two meta-analyses in this review, and both of them showed a trend towards a decline in verbal fluency [26,27]. While Xie et al. discovered worsening in other cognitive domains, like memory and executive functioning as well, Martinez et al. did not get worsening in such domains [26,27]. 

*Changes in Attention/Memory* 

The existing heterogeneity among the tests for attention/memory has led to variability in result presentation and compilation. Troster et al. discovered a small improvement on the Wechsler memory scale, third edition, abbreviated (WMS-III-A) subtest score (immediate and delayed recall of picture scenes and stories) in the stimulation group after 90 days but by using alternative testing technique no such increments were observed. Hence, the increment could be attributed to the practice effect in the study group [17]. A decline in attention found out by worse performance in all portions of the Stroop task except for interference [17]. Still, conflicting results were presented in the RCT by Witt et al., who said the worsening in the Stroop task was most prominent in the interference effect [19]. No decline in the verbal memory (by Ray's auditory verbal learning test scores) was present. Tramontana et al. saw similar declines in attention; after 12 months, the difference in Wechsler adult intelligence scale-III (WAIS-III) digit span (p = 0.004) and preservative error portion of Wisconsin card sorting test (WCST) (p = 0.053) were statistically significant between the two comparison groups [16]. In the study by Zangaglia et al., worsening noticed in WCST scores at one month, post-operative returned to baseline after 12 months and stayed the same. This decline in cognitive flexibility was only transient [21]. Saez-Zea et al. found a slightly lesser deterioration in the time taken to complete Trail making test-B for attention in the DBS group. They attribute the result to the confounding effect of the lower mean age of patients in the DBS group. Overall, there was no significant decline in executive functions [22]. Fasano et al. described a decline in delayed memory recall (Rey auditory verbal learning test, RAVLT, scores) and cognitive flexibility (WCST scores) after eight years in all the patients, but it was mild and clinically insignificant in 13 of the 16 patients [25].

Outcome Predictors

Risk factors predicting the development of cognitive decline, although defined previously, are still unknown. Smeding et al. discovered that the test scores for attention, age at surgery, and levodopa response were the best predictors of multivariate-cognitive decline and that an umbrella of baseline patient and disease-related characteristics must be considered in post-op neuropsychological evaluation [30]. A positron emission tomography (PET) study of nine STN-DBS patients at six months revealed decreased metabolic activity in the left dorsolateral prefrontal and Broca cortical areas in the three patients whose verbal fluency was impaired [31]. Concerning the acute effects of electrical stimulation, a PET study of regional cerebral blood flow in seven patients showed greater activation of a left-sided fronto-temporal network and the right frontal cortex during a verbal fluency task when stimulation was off and lesser activation when stimulation was on. They suggested that interference with the basal ganglia-thalamus-prefrontal cortex circuit may underlie the verbal fluency deterioration [32]. Another factor could be a disruption by chronic stimulation of the dense neuronal fibers present between the STN and cognition controlling regions such as limbic and association areas [27].

Limitation

Only a couple of the studies collected have made cognition their primary outcome, as most of the studies were focused on the motor outcomes. A lack of standardized testing domain could be the reason for the variability of the cognitive results. Another limitation was the exclusion of studies in languages other than the English, which might have created an unintentional bias. Our review was solely focused on the patients with STN implants, and comparison with PD patients with implantation in the GPi could provide better insight into the role structural irritation plays in cognitive decline.

## Conclusions

STN-DBS is the gold standard procedure for patients with PD in motor outcomes but produces unpredictable cognitive outcomes. Through the medium of this review, we gathered that there is an appearance of minor but pertinent cognitive side-effects in terms of executive dysfunction and decreased attention. The decline in verbal fluency was most prominent and could be explained by alteration in the neuronal connections surrounding the implant. Such changes were small and did not impact the QOL, a factor which improved significantly in most of the patients attributing to the medication reduction and favorable motor functioning. Nevertheless, these small changes could have an additive effect in the long term, and understanding the mechanism behind these changes could help prevent them in the future. All but two studies followed the patients for a short span of one year following surgery. Thus, creating a need for more comprehensive long-term controlled trials focused solely on the impact on cognition comparing different baseline characteristics of the patients with PD. At the same time, while analyzing, we observed a slight trend towards increased adverse outcomes after the surgery and in some probability of psychiatric comorbidities like depression and apathy. Whether these changes occur as a result of disruption of surrounding areas of the brain or there is some correlation of such adverse effects with the executive dysfunctions needs more analysis.
